# A Systematic Critical Appraisal for Non-Pharmacological Management of Osteoarthritis Using the Appraisal of Guidelines Research and Evaluation II Instrument

**DOI:** 10.1371/journal.pone.0082986

**Published:** 2014-01-10

**Authors:** Lucie Brosseau, Prinon Rahman, Karine Toupin-April, Stéphane Poitras, Judy King, Gino De Angelis, Laurianne Loew, Lynn Casimiro, Gail Paterson, Jessica McEwan

**Affiliations:** 1 School of Rehabilitation Sciences, Faculty of Health Sciences, University of Ottawa, Ottawa, Ontario, Canada; 2 Department of Community Health and Epidemiology, Dalhousie University, Halifax, Nova Scotia, Canada; 3 Children's Hospital of Eastern Ontario Research Institute, Ottawa, Ontario, Canada; 4 Department of Pediatrics, Faculty of Medicine, University of Ottawa, Ottawa, Ontario, Canada; 5 Department of Academic Affairs, Montfort Hospital, Ottawa, Ontario, Canada; 6 The Arthritis Society, Ottawa, Ontario, Canada; 7 University of Ottawa Library, Ottawa, Ontario, Canada; Universidad Peruana de Ciencias Aplicadas (UPC), Peru

## Abstract

Clinical practice CPGs (CPGs) have been developed to summarize evidence related to the management of osteoarthritis (OA). CPGs facilitate uptake of evidence-based knowledge by consumers, health professionals, health administrators and policy makers. The objectives of the present review were: 1) to assess the quality of the CPGs on non-pharmacological management of OA; using a standardized and validated instrument - the Appraisal of Guidelines Research and Evaluation (AGREE II) tool - by three pairs of trained appraisers; and 2) to summarize the recommendations based on only high-quality existing CPGs. Scientific literature databases from 2001 to 2013 were systematically searched for the state of evidence, with 17 CPGs for OA being identified. Most CPGs effectively addressed only a minority of AGREE II domains. Scope and purpose was effectively addressed in 10 CPGs on the management of OA, stakeholder involvement in 12 CPGs, rigour of development in 10 CPGs, clarity/presentation in 17 CPGs, editorial independence in 2 CPGs, and applicability in none of the OA CPGs. The overall quality of the included CPGs, according to the 7-point AGREE II scoring system, is 4.8±0.41 for OA. Therapeutic exercises, patient education, transcutaneous electrical nerve stimulation, acupuncture, orthoses and insoles, heat and cryotherapy, patellar tapping, and weight control are commonly recommended for the non-pharmacological management of OA by the high-quality CPGs. The general clinical management recommendations tended to be similar among high-quality CPGs, although interventions addressed varied. Non-pharmacological management interventions were superficially addressed in more than half of the selected CPGs. For CPGs to be standardized uniform creators should use the AGREE II criteria when developing CPGs. Innovative and effective methods of CPG implementation to users are needed to ultimately enhance the quality of life of arthritic individuals.

## Introduction

Osteoarthritis (OA) is known as a degenerative disorder of the joint cartilage associated with hypertrophic bone changes [1] and it is recognized as the most common chronic joint disease in the world [2]. It is expected that OA will be the fourth leading cause of disability by 2020 and the 6th leading cause of years lived with disability [3]–[4]. The annual absenteeism costs related to OA in North America are $10.3 billion [5]. The management of OA in patients should be comprehensive and should target pain reduction, improvement and maintenance of joint function, a decrease in disability, and education of parents about disease and therapies [6]. While people with severe and persistent OA symptoms may use pharmacological treatments such as nonsteroidal anti-inflammatory drugs (NSAIDs), cyclo-oxygenase-2 (COX 2) inhibitors, and undergo joint arthroplasty [7]–[9], people with mild to moderate OA symptoms should consider conservative management by combining pharmacological and non-pharmacological interventions [7], [10]. Non-pharmacological interventions are essential to the treatment and management of any chronic disease and they are as important as pharmacological interventions [6]. According to Sakalauskiene (2010) [6], most non-pharmacological interventions 1) are low in cost; 2) incorporate self-management performed at home or in the community; and 3) have a substantial public health impact. Non-pharmacological interventions, such as therapeutic exercises and weight control, have been shown to be effective in reducing pain and improving function in OA and are usually safe [6]–[7]. However, their use is often suboptimal, which warrants further knowledge translation to clinicians and patients about their importance in improving health outcomes [11]–[12]. Numerous clinical practice CPGs (CPGs) exist in rheumatology, which are intended to facilitate knowledge translation to clinicians and evidence-based clinical decision making. In order to make optimal and accurate clinical decisions for their arthritic patients, health professionals should use high-quality CPGs. In previous systematic reviews [13]–[16], CPGs that considered non-pharmacological and pharmacological interventions have been appraised. However, the CPGs which targeted only non-pharmacological interventions have never been assessed with the Appraisal of Guidelines Research and Evaluation II (AGREE II) tool [14]–[15]. Our paper focused on the quality assessment of non-pharmacological interventions, especially in terms of rigour of development. Non-pharmacological interventions include electrotherapy (e.g. transcutaneous electrical nerve stimulation), patient education, team approach (e.g. multidisciplinary team approach), therapeutic exercises (e.g. aquatics), weight management and other interventions (e.g. acupuncture assistive devices, etc.) [17]–[18]. This review will provide health care professionals a platform to compare the development of recommendations for non-pharmacological interventions from the AGREE II scoring. It will also help them accept and implement the recommended interventions in their health practice. The objective of this critical appraisal review is to 1) identify the CPGs focusing on non-pharmacological management of OA in all joints of the upper and lower extremities; 2) assess the quality of the CPGs using the updated AGREE II instrument (www.agreetrust.org); and 3) to document the non-pharmacological recommendations and identify the high quality CPGs.

## Methods

The systematic review of CPGs used the Cochrane Methodology (www.cochrane.org) to identify, select and analyze the data and the PRISMA statement to guide the reporting of the systematic review [19] (Appendix S1). Ethics approval was not required, as this work was based on a systematic literature review.

### Literature search

A systematic literature search was performed by an experienced librarian (JM) using relevant key words (Appendix S2) in specific databases: AMED, CINHAL Medline, Embase (Appendix S2). In addition, a hand search was also performed from two main sources: 1) existing guideline inventories and guideline homepages, such as PEDro (http://www.pedro.fhs.usyd.edu.au/index.html) [20], the National Guideline Clearinghouse (http://www.guideline.gov/) [21], Guideline International Network (http://www.g-i-n.net/) Turning Research into Practice (TRIP) (http://www.tripdatabase.com/) [23] 2) the reference lists found in the selected/included CPGs.

### Eligibility criteria

In order to be inclusive of a wider range of non-pharmacological interventions, CPGs were included if they were current (2001–2013), consisted of a non-pharmacological component for adult patients (≥18 years of age) with osteoarthritis, and written in English. All CPGs considered included a grading system to evaluate the evidence and have been peer-reviewed prior to publication. Two pairs of independent evaluators assessed the eligibility of all potential CPGs and were considered if they fulfilled the inclusion criteria (Appendix S3) established prior to the literature search.

### Data extraction procedures

The quality of each CPG was assessed using the AGREE II instrument (www.agreetrust.org). The AGREE II is a 23-item instrument which consists of six domains evaluating different aspects of CPG development: 1) scope and purpose: the objective of the guideline, the target population and health question; 2) stakeholder involvement: involvement of stakeholders in the guideline development process and patients views and preferences 3) rigour of development: the process to collect and synthesize evidence and the recommendation development process; 4) clarity and presentation: the language, structure and the presentation of the guideline; 5) applicability: looking at the barriers and facilitators for implementation, approach to improve uptake; and 6) editorial independence: identifying biases resulting from competing interests. The AGREE II uses a 7-point Likert scoring system ranging from 1 (strongly disagree), which corresponds to an item very poorly reported to 7 (strongly agree), which means an exceptional quality of reporting. A scoring from 2 to 6 was assigned if all the items in the domains were not considered; the scoring would increase depending on the consideration and fulfilment of each criterion (www.agreetrust.org).

### Assessment of the methodological qualities of CPGs

Two pairs of evaluators with AGREE II experience independently assessed the selected CPGs with the AGREE II instrument and to prevent a potential information bias a third experienced evaluator was involved when a co-author of this review was a co-creator of an included CPG (Table 1). All three evaluators received training from the online tutorial and practice guideline (http://www.agreetrust.org/resource-centre/training/). For each CPG, the items from AGREE II instrument(http://www.agreetrust.org/wp-content/uploads/2013/06/AGREE_II_Users_Manual_and_23-item_Instrument_ENGLISH.pdf) was completed which included all relevant information and ratings on the 7-point scale for all six domains. The total scores were computed using the McMaster calculator (). The inter-rater agreement for AGREE II scores was also computed and a discussion about scoring occurred when the standard deviation (SD) between the two evaluators for each domain was greater than 1.50. The AGREE II consortium has not set a minimum or maximum range for domain score quality; therefore, it is difficult to differentiate between high and low quality CPGs. We used the criteria of previous guideline appraisals [14], [24], where domain scores greater than 60% are considered effectively addressed. The guideline is recommended and considered high quality based on the rigor of development and if three or more domains were effectively targeted (greater than 60%) [24].

**Table 1:** The Included CPGs.

Clinical Practice Guideline	Hand Management	Hip Management	Knee Management
AAOS [26]			✓
ACR [27]	✓	✓	✓
Eular [28]–[30]	✓	✓	✓
Eular [31]		✓	✓
Kjeken [32]	✓		
NICE [33]		✓	✓
OARSI [34]		✓	✓
Ottawa Panel [35]–[39]	✓	✓	✓
Peter et al. [42]		✓	✓
Philadelphia Panel [40]–[41]			✓
RACGP [44]		✓	✓
Roddy et al. [43]		✓	✓

ACR: American College of Rheumatology; EULAR: The European League against rheumatism; NICE: National Institute for health and Clinical Excellence; OARSI: Osteoarthritis Research Society International; PGrip: People Getting a Grip on Arthritis RACGP: Royal Australian College of General Practitioners.

### Strength of recommendations for each non pharmacological interventions in the CPGs

In addition to assessing the methodological quality, the evaluators compiled all the non-pharmacological interventions assessed in at least one of the included CPGs (Table S1). The strength of the recommendations related to the interventions was translated into a 4-point hierarchical category system: 1) strongly recommended; 2) recommended; 3) weak evidence; and 4) insufficient evidence. Each of these categories was adjusted for equivalence and uniformity according to the individual scoring system of each of the seventeen CPGs included in this review. The grade “strongly recommended” corresponds to the highest grading and is usually based on at least one high-quality systematic review or randomized controlled trial (RCT) and represents a strong body of evidence. The grade “recommended” corresponds to a CPG grading usually based on at least one controlled clinical trial (CCT) or RCT of lower quality, and represents a good body of evidence that can be trusted to guide practice in most situations. “Weak evidence” is based on a strong clinical or expert opinion based on current practice, without the support of scientific evidence, and needs to be applied with caution. “Insufficient evidence” is assigned when there is a lack of scientific results or conflicting results not supported by clinical or expert opinion.

### Inter-rater reliability study

The inter-rater reliability study for AGREE II was conducted to ensure the reliability of the CPGs quality scores between pairs of evaluators. Each individual evaluator completed the quality appraisal evaluation to determine the quality of each recommendation statement. The sub-total scores obtained by one evaluator for each domain were compared with those rated by the second evaluator. The statistical analysis involved intraclass correlation coefficients (ICCs) based on an ANOVA (random) procedure for repeated data [25]. The analysis of the reliability study was performed with Statistical Package for Social Sciences (SPSS) version 20.

## Results

### Search results

A systematic search provided a total of 1136 citations. Figure S1 provides a flow diagram of how the included CPGs were selected.

### Characteristics of studies

A total of 17 CPGs [26]–[44] were included for appraisal according to selection criteria and all of the included CPGs provided recommendation for the management of hand, hip or knee (Table 1). Out of the 17 CPGs, 9 CPGs [31]–[32], [35]–[42], [44] were considered non-pharmacological CPGs exclusively and 8 CPGs [26]–[30], [33]–[34], [43] included both pharmacological and non-pharmacological interventions (Table 2).

**Table 2:** CPGs that considered pharmacological and non-pharmacological interventions.

CPGs that considered pharmacological+non-pharmacological interventions	CPGs that only considered Non-pharmacological interventions.
AAOS [26]	Eular [31]
ACR [27]	Kjeken [32]
Eular [28]–[30]	Ottawa Panel [35]–[39]
NICE [33]	Philadelphia Panel [40]–[41]
OARSI [34]	Peter et al. [42]
RACGP [44]	Roddy et al. [43]

American College of Rheumatology; EULAR: The European League against rheumatism; NICE: National Institute for health and Clinical Excellence; OARSI: Osteoarthritis Research Society International; PGrip: People Getting a Grip on Arthritis RACGP: Royal Australian College of General Practitioners.

### The AGREE II quality scores of the studies

The quality scores for each domain of the AGREE II instrument for the 17 included CPGs [26]–[44] are found in table 3 and the individual scores for each item can be found in appendix S4. The methodological quality ranged from 45%–80% for the various CPGs, domain 1 being of highest quality and domain 5 being of lowest quality (Table 3). D1: *scope and purpose* had a mean score of 70%±20% for the 17 [26]–[44] included CPGs and both the Philadelphia Panel [38]–[39] and the series of Ottawa Panel CPGs [35]–[39] scored the highest at 97%. D2: *stakeholder involvement* obtained a lower mean score of 61%±21%. The National Institute of for Health and Clinical Excellence for the Care and Management of Osteoarthritis (NICE) [33] had the highest score at 89% and the series of EULAR [28]–[30] CPGs scored the lowest for this domain with 28%. Domain 3: *rigor of development* had a mean score of 63%±11%. American Academy of Orthopaedic Surgeons [26] scoring the highest for this domain at 80% and out of the 17 CPGs, AAOS [26] underwent the most extensive development process. Domain 4: *clarity of presentation* scored a mean of 86%±9.1% with Peter et al CPG scoring the highest at 97%. Domain 5: *applicability* had a poor mean score of 14%±16%, for this domain three CPGs [26], [32], [44] obtained 0%. Domain 6: *editorial independence* also had a poor mean score at 31%±19.1%.

**Table 3:** Quality Scores using AGREE II Instruments for included CPGs on OA.

Agree II domains	AAOS	ACR	Eular	Eular	Kjeken et al.	NICE	OARSI	Ottawa Panel	Peter et al.	Philadephia Panel	RACGP	Roddy et al.	(mn ±SD)
	[26]	[27]	[28]–[30]	[31]	[32]	[33]	[34]	[35]–[39]	[42]	[40]–[41]	[44]	[43]	
Domain 1	78%	89%	31%	56%	56%	67%	56%	97%	56%	97%	81%	75%	70%±20%
Domain 2	61%	72%	28%	78%	19%	89%	67%	72%	67%	67%	75%	39%	61%±21%
Domain 3	80%	67%	54%	57%	52%	70%	78%	65%	45%	70%	62%	55%	63%±11%
Domain 4	89%	92%	89%	89%	86%	81%	89%	61%	97%	78%	89%	89%	86%±9%
Domain 5	0%	10%	17%	0%	0%	56%	27%	10%	0%	25%	21%	0%	14%±16%
Domain 6	67%	29%	8%	13%	33%	25%	67%	42%	21%	17%	21%	33%	31%±19%
Quality of CPGs (mn±SD)	5.5±0.71	4.5±0.71	4±0	4.5±0.71	4±0	5.5±0.71	4±0	5.5±0.71	4±0	5±0	6±1.41	5±0	4.8±0.41
Assessed by	PR & KTA	PR &LB	PR & KTA	PR & KTA	PR & KTA	PR & KTA	PR & KTA	PR & KTA	PR & KTA	PR & KTA	PR & KTA	PR & KTA	PR & KTA

Domain 1. Scope and Purpose; Domain 2: Stakeholder involvement; Domain 3: Rigour of Development; Domain 4: Clarity of Presentation; Domain 5; Applicability Domain 6: Editorial Independence; Quality of CPGs: Using AGREE II scoring system :1–7; ACR: American College of Rheumatology; EULAR: The European League against rheumatism; NICE: National Institute for health and Clinical Excellence; OARSI: Osteoarthritis Research Society International; PGrip: People Getting A Grip on Osteoarthritis; RACGP: Royal Australian College of General Practitioners; mn: mean; SD: standard deviation.

Overall, ten [26]–[27], [33]–[41], [43] out of the seventeen CPGs were deemed high quality (>60%) based on rigour of development and effectively targeting 4–5 out of the 6 domains, one of the CPGs [26] was able to effectively target five out of the six domains and the remaining CPGs [27], [33]–[41], [43] effectively targeted four domains out of the six. *Clarity of presentation* was the domain that was most effectively addressed by all of the CPGs and *applicability* was the most poorly addressed domain. None of the CPGs effectively addressed domain 5 and CPG scores ranged from 0%–56%. Only a minority of the domains were effectively targeted by most of the CPGs (domain 1, 2, 3 & 4). None of the included CPGs effectively targeted all six of the domains.

Interestingly, out of the nine CPGs that solely looked at non-pharmacological interventions (Table 2), only the Philadelphia Panel [40]–[41] and the series of Ottawa Panel CPGs [35]–[39] are considered to be a high quality guideline (>60%). Roddy et al [43], EULAR [31] & Peter et al [42] only effectively targeted two out of the six domains and Kjeken et al [32] effectively targeted only one out of the six domains.

For CPGs that targeted pharmacological interventions and non-pharmacological interventions (Table 2), five [26]–[27], [33]–[34], [43] out of the eight CPGs were deemed high quality and effectively addressed four to five domains. All five CPGs scored high for two domains in particular: *Scope and purpose and clarity of presentation*. Consequently, all five CPGs [26]–[27], [33], [35] were recommended for practice. The series of EULAR [28]–[30] CPGs were the only CPGs which targeted both pharmacological and non-pharmacological interventions and not graded as a high quality CPG with a low score of 4/7 for overall quality of the CPG. Both appraisers recommended this guideline for practice, but with modifications.

When asked to rate the overall quality of the CPGs, ten [26]–[27], [31], [33], [35]–[41], [43]–[44] out of the seventeen were recommended for practice by both appraisers and seven CPGs [28]–[30], [32], [34], [42], were recommended but with modifications (Appendix S4).

### The strength of the recommendations

The strength of the recommendations for each intervention was provided by the developers of the included CPGs [26]–[44] (Table S1). The recommendations formulated by these CPGs were categorized by alphabetical order: 1) Electrotherapy; 2) Other Interventions; 3) Patient Education; 5) Team Approaches; 6) Therapeutic Exercises; 7) Weight Management (Table S1). Generally speaking, the strength of the recommendation varied largely amongst the 17 CPGs. Table S1 summarizes the strength of all the recommendations of the included CPGs according to interventions.

#### Electrotherapy

Transcutaneous electric nerve stimulation (TENS) were recommended by the majority of the included CPGs. Ten [27], [28]–[30], [34]–[41] out of the seventeen CPGs recommended or strongly recommended TENS. Only one CPG [43] found insufficient evidence to recommend TENS.

#### Other interventions

Acupuncture was recommended or strongly recommended by 9 CPGs [27], [28]–[30], [33]–[40] and 2 CPGs [26], [43] found insufficient evidence to recommend it for the management of osteoarthritis. Foot orthoses and insoles were recommended or strongly recommended by ten CPGs [27]–[30], [34]–[39]. Again for heat/cryotherapy a total of twelve [27]–[30], [33], [35]–[39], [42]–[43] out of seventeen mentioned this intervention in their CPGs and ten [27]–[30], [33], [35]–[39] recommended it and two [42]–[43] found insufficient evidence. Patellar taping was recommended in six CPGs [26]–[30], [42]; However one [44] CPG found insufficient evidence to recommend this intervention.

#### Patient education

Patient education combined with exercises was strongly recommended by nine CPGS [38]–[40], [34]–[39] out of the seventeen CPGs and recommended by five CPGs [26]–[27], [41], [33], [42].

#### Team approach

Only one guideline [44] mentioned multidisciplinary approach and physiotherapy as an intervention. However, the guideline found weak evidence to recommend it for the management of OA.

#### Therapeutic exercises

For therapeutic exercises the two interventions recommended by the majority of the CPGs were aerobic exercises, behavioral approach combined with therapeutic exercises and strengthening exercises. Aerobic exercises were recommended by thirteen [26]–[30], [34]–[39], [44] out of the seventeen CPGs, Behavioral approach combined with therapeutic exercises was recommended by eight [27], [31], [35]–[39], [44] out of seventeen and strengthening exercises were recommended by nine [27], [31], [32], [35]–[41].

#### Weight management

Control of weight was recommended or strongly recommended by thirteen [26], [27], [28]–[30], [33]–[39], [44] of the seventeen CPGs and only four CPGs [35]–[39] strongly recommended diet alone, diet combined with physical activity as well as physical activity as a sole intervention.

### Inter-rater reliability study

The AGREE II scores exhibits an overall very good inter-rater reliability with an ICCs values ranging from 0.86 (good reliability) to 0.95 (high reliability) depending on the domain assessed (Table 4). These results indicate the AGREE II quality scores of the included CPGs obtained between pairs of evaluators are reliable (Table 4).

**Table 4:** Inter rater reliability study results for included CPGs.^a^

Agree II domains	P(G)MS^b^	RMS	EMS	*n*	K	ICC (Random)	Lower 95% Cl	Upper 95% Cl	Pvalue
Domain 1	26.1	18.4	3.6	17	2	.86 (good)	.52	.96	.001
Domain 2	24.2	8.2	3.2	17	2	.87 (good)	.55	.96	.001
Domain 3	65.2	60.2	8.5	17	2	.87 (good)	.55	.96	.001
Domain 4	5.4	0.042	.68	17	2	.88 (good)	.56	.96	.001
Domain 5	32.8	6.0	2.5	17	2	.92 (high)	.73	.98	.00
Domain 6	8.7	.38	.47	17	2	.95(high)	.81	.99	.00

Domain 1: Scope and Purpose; Domain 2: Stakeholder involvement; Domain 3: Rigour of Development; Domain 4: Clarity of Presentation; Domain 5; Applicability Domain 6: Editorial Independence;

^a^ CPGs = clinical practice CPGs; P(G)MS = Patients' (Guideline's) Mean Square; RMS = Rater's Mean Square; EMS = Error Mean Square; n = sample size; K = number of measurements; ICC = intraclass correlation coefficient; CI = confidence interval.

^b^ Temporary PMS, RMS, and EMS values.

## Discussion

This review identified a total of 17 CPGs on the non-pharmacological management of OA in all joints of the upper and lower extremities. Among the 17 CPGs considered, nine of these solely focus on non-pharmacological interventions and the remaining eight comprise a combination of pharmacological and non-pharmacological interventions. According to the AGREE II instrument, ten CPGs [26]–[27], [33]–[41], [44] were recognized as good quality CPGs with high scores for rigor of development and because they effectively targeted four to five domains. Therapeutic exercises, patient education, Transcutaneous Electrical Nerve Stimulation, acupuncture, orthoses and insoles, heat and cryotherapy, patellar tapping and weight control are commonly recommended for the non-pharmacological management of OA by the high-quality CPGs. It was noted that common recommendations were found by the majority of the CPGs; however, the strength of the recommendations varied between the CPGs.

In the past, OA CPGs have been evaluated with the AGREE I instrument [13]–[14], [16], [45]. To our knowledge, the AGREE II instrument has been used to appraise pharmacological and non-pharmacological CPGs for the management of OA; however, no quality scoring was presented in the literature [15]. Therefore, the results of this review can only be compared partially with previous literature for CPGs on non-pharmacological and pharmacological interventions.

### Assessment of the quality for the included CPGs

#### Domain 1: Scope and purpose

All the included CPGs obtained a high (>60%) AGREE II quality score for domain 1 (scope and purpose), except for Peter et al. [42], Kjeken et al. [32] & OARSI [36] CPGs and all three CPGs were not able to effectively target this domain at 56%. The score of 56% was due to failure to provide detailed descriptions of the health question covered by the CPGs (item 2) and the target population (item 3). For a guideline to obtain a high score for item 2, the Population Intervention Comparison Outcome PICO model should be used when describing the health question(s) covered [15], [46]–[47]. The series of Ottawa [32]–[36] and the Philadelphia panels [37] CPGs were the only ones that described the population using the PICO model [46]. Finally, although the CPGs did provide the overall objective, sufficient details about the objective were not provided to the readers [15]. These results are consistent with previous reviews conducted with the AGREE I instrument on OA CPGs [13]–[14], [45], where the scope and purpose was effectively addressed by a majority of the OA CPGs

#### Domain 2: Stakeholder Involvement

All the CPGs, except for EULAR [28]–[30], Roddy et al. [43] & Kjeken et al. [32], described the stakeholder involvement. The Kjeken et al. [32] CPG was targeted to occupational therapists, but the CPG lacked credentials on the professionals who were involved in the development process. In addition, both EULAR [28]–[30] and Roddy et al. [44] were graded low for items 5 & 6, as they did not clearly define the target users of the CPG. Although the majority of the CPGs provided information about the development groups, most did not provide details on whether patient preferences and views were considered during the recommendation development phase [15], [26]–[44]. The series of Ottawa Panel [32]–[39], NICE [33], RACGP [44] and EULAR [31] CPGs were the only ones which considered patient views and preferences during the development phase of the CPGs. Both the series of Ottawa Panel Guideline [35]–[39] and EULAR [31] CPGs chose patients with OA to be part of the panel. The NICE [33] guideline consisted of a patient with OA, a consumer expert, researcher and organization representatives on the guideline development group and the RACGP [44] had patient representatives in the RACGP [44] working group [15], [33], [35]–[39], [44]. Previous reviews by Poitras et al. [14], Misso et al [13] and Penchaz et al. [47] with the AGREE I also had a significant low quality score for this domain as a result of the majority of the CPGs not reporting seeking patient views and preferences.

#### Domain 3: Rigour of development

This domain obtained a relatively low scoring for the majority of the CPGs, but more specifically for Kjeken et al. [32]; EULAR [28]–[31] and Roddy et al. [43]. For all of the CPGs considered, systematic literature searches were conducted and the evidence found was rated using a grading system [15], [26]–[44]. Some of the CPGs provided search strategies in the document (i.e. appendix and reference) [26]–[44] and a few provided online links to the literature [27], [33], however the links were not functioning. The grading systems differed for each CPG. Peter et al. [42] used the EMBRO system; EULAR [28]–[31], Kjeken et al. [30] and Roddy et al. [43] used an evidence hierarchy; ACR [27] used the GRADE system (www.cochrane.org); RACGP [44] used the SIGN appraisal tools and NHMRC assessment; NICE [33] used the NICE method; AAOS [26] used AMSTAR and evidence hierarchy; OARSI [34] used Oxman and Guyatt and the Jadad scale, and the series of Ottawa Panel CPGs [35]–[39] used the Jaded scale. Kjeken et al. [32], EULAR [28]–[30] and Roddy et al. [43] all received a score lower than 60% ranging from (45%–57%). These CPGs had insufficient information on how the body of evidence was evaluated for bias (item 9), the strengths and limitations of the body of evidence (item 9), how the development process influenced the recommendations (item 10), and a procedure for updating the guideline (item 14). Since a majority of the CPGs failed to provide detailed information on item 14 for whether it would be updated and/or the updating procedures, this resulted in an overall lower score in domain 3. This review's results are consistent with previous reviews on appraisals with AGREE I [13]–[14], [43]. A majority of the CPGs provided recommendations with evidence, highlighted key recommendations, and considered health benefits and harm when formulating the recommendation. However, the mean quality score for rigor of development from AGREE I for the two previous reviews was found to be 47% (13) and 43% (14) significantly lower than the mean of 63%±11% based on the AGREE II instrument. A rationale for the difference in scoring can be attributed to using a rating scale from 1–7 as opposed to 1–4 in AGREE I instrument.

#### Domain 4 (clarity of presentation)

All the CPGs effectively addressed this domain and most received a relatively high score (75%–97%). The NICE [28] and the RACGP [37] CPGs consisted of clinical algorithms, which may facilitate clinical decision making [7], [15]. Physiotherapy (PT) and occupational therapy (OT) are rehabilitation professions which provide a large spectrum of non-pharmacological interventions to patients with OA. Consequently more precision about PT and OT treatment modalities needs to be provided, especially regarding specific therapeutic interventions; CPGs should not refer to the professions of PT and OT as interventions. However, the AGREE II scores are consistent with previous reviews [13]–[14], [43], domain 4: clarity and presentation was the most effectively addressed out of all the domains.

#### Domain 5 (applicability)

This domain obtained the lowest AGREE II scores for all the CPGs on OA, as most CPGs only describe a pilot study without providing results, or do not mention an implementation strategy besides dissemination or publication. Compared to all of the other CPGs, the NICE [31] guideline scored the highest as it provided an implementation section which consisted of algorithms and online resources for stakeholders [31]. None of the CPGs effectively addressed all of the items in this domain. Reviews conducted by Poitras et al. [14], Misso et al. [13], and Penchaz et al. [43] also found a clear weakness for this domain, as a majority of the CPGs failed to provide a strategy of dissemination and implementation. In addition, this domain also had the lowest scoring for all three reviews [13]–[14], [45].

#### Domain 6 (Editorial independence)

None of the CPGs effectively addressed all of the items for this domain. While many of the CPGs mentioned the funding body, very few were able to provide information on competing interests of the development group except AAOS [26] and OARSI [34]. While many of the CPGs mentioned the funding body, very few failed to provide information on competing interests of the development group. Similar to the results, the reviews conducted by Poitras et al. [14], Misso et al. [13], and Penchaz et al. [45] found this domain to be poorly addressed.

### Strength of recommendations

A common theme found when assessing the quality of the CPGs was that the majority of interventions, with the strongest evidence, looked at both pharmacological and non-pharmacological interventions [13]–[14], [45]. However, minority interventions or infrequently used interventions were considered as recommendations in CPGs that only looked at non-pharmacological interventions. The series of Ottawa Panel CPGs [35]–[39] was an exception where both the common and infrequent interventions were addressed.

### Implications of score

There is existing high-quality CPGs for the non-pharmacological management of mild to moderate OA. However, the dissemination as well as the implementation of non-pharmacological CPGs in rheumatology is a challenging issue. This is also reflected in the CPG development, and observed with the lower ratings (0%–56%) obtained for applicability of AGREE II (domain 5) for the 11 CPGs included in this review (Table 3). This observation was made over the years regarding the low application rate of non-pharmacological interventions among health professionals in rheumatology [6]. Indeed, family doctors and rheumatologists [48]–[53] prescribed less than 50% (30%–45%) physical interventions, not necessarily recommended, such as aerobic and range of motion exercises, orthoses, and assistive devices for ambulation, acupuncture, and OT or energy conservation. Surprisingly, very low medical referral rates are observed for patient education (29%), as well as for arthritis self-management programs (3%) [48], although these interventions are recommended by most CPGs (Table S1). Furthermore, individuals with arthritis adopted non-pharmacological interventions more frequently by themselves than prescribed by health professionals [49].

### Limitations

The AGREE II instrument is intended to be an improved version of the original AGREE I instrument [11]–[12]. The AGREE II instrument was available in 2009 with validation studies conducted in 2010. [54]–[55]. Despite this update, more recent CPGs published after this date [31]–[32], [42], [44] did not benefit from the improved instrument in their overall quality assessment. This is found for items in domain 5 (applicability), where more detail is required for tools, facilitators, and barriers for application; and domain 6 (editorial independence), where CPGs must provide details on the description and methods by which potential competing interests were identified.

The AGREE II instrument focuses on the methodological process for guideline development in domain 3. Although this is important to consider, the scorings received in domain 3 do not ensure that the recommendations are valid because the process to develop the guideline does not equate with its quality [56]. For example, the AGREE II assesses in domain 3 if the literature for the CPG was systematically searched, but fails to consider if the recommendations of each intervention were analyzed using a quantitative approach. Domain 3 also failed to consider if the primary comparative controlled trials underlying the CPGs have high-quality standards. The studies included in the CPGs should be assessed by instruments such as Jadad [57] and Pedro scale [22], [58] for CCSs and Amstar [59] for systematic reviews.

The grading system and the potential subjectivity could lead to question interpretation and could be considered as a weakness of the AGREE II. There is a user manual for AGREE II which the appraisers can use to evaluate the CPGs; however, when only some of the criteria for an item are met the appraiser must use their best judgment to score the item.

The weight of each item per domain is also problematic. This was especially seen with the scoring for domain 3, where all six items for the domain were regarded equally and as a result the majority of the CPGs received a lower score for this domain. Thus, even if the CPG received higher scores and effectively addressed the other items (i.e. the systematic methods used, criteria for selecting the evidence, method for formulating the recommendations etc.), the overall quality percentage was deemed low because they failed to address item 14 (a procedure for updating the guideline). This was especially seen with the series of Ottawa Panel CPGs [35]–[39]. This guideline received high scores for the five out of the six items in domain three, but only scored 65% on the overall quality of rigor of development.

The 7-point scale used in the AGREE II instrument is based on the idea that if all the elements of a particular item are fully addressed, then it is given a score of seven for that particular item. Conversely, if none of the elements were present then it was given a score of 1. An initial score of 1 (absence of information) is considered a systematic error, because AGREE II does not consider “not applicable” as a response according to its scoring system.

There is a potential publication bias, as only CPGs published in English were chosen according to the selection criteria. In addition, the recommendations based on the CPGs are difficult to establish because there is a contradiction between the strength of the recommendation. CPGs often referencing the same non-pharmacological studies will grade the strength differently, ranging from insufficient evidence to strongly recommended. A rationale for this inconsistency could be attributed to the panel's difficulty in dealing with conflicting data from primary methods. A technique to address this is to engage in a quantitative method such as the Cochrane Collaboration methodology to resolve conflicting results between two RCTs.

## Conclusion

From the total seventeen CPGs included, based on the AGREE II scoring, we found ten good quality CPGs [26]–[27], [33]–[41], [44] where the rigor of development was >60%. There are good-quality CPGs available for health professionals. There was consensus for some of the recommendations, such as therapeutic exercises, patient education, Transcutaneous Electrical Nerve Stimulation, acupuncture, orthoses and insoles, heat and cryotherapy, patellar tapping and weight control for the management of OA.

## Supporting Information

Table S1 **Recommendations for the Management of Osteoarthritis.** ACR: American College of Rheumatology; EULAR: The European League against rheumatism; NICE: National Institute for health and Clinical Excellence; OARSI: Osteoarthritis Research Society International; PGrip: People Getting a Grip on Arthritis RACGP: Royal Australian College of General Practitioners TENS: Transcutaneous Electric Nerve Stimulation.
(DOCX)

Figure S1 **Prisma flow diagram of included CPGs.**
(DOC)

Appendix S1 **Prisma 2009 Checklist.**
(DOC)

Appendix S2 **Literature Search Strategy.**
(DOCX)

Appendix S3 **The Eligibility Criteria.**
(DOCX)

Appendix S4 **Raw Data.**
(DOCX)
