# Supplementary material for: A Systematic Critical Appraisal for Non-Pharmacological Management of Osteoarthritis Using the Appraisal of Guidelines Research and Evaluation II Instrument
Source: PLoS One. 2014 Jan 10;9(1):e82986. doi: 10.1371/journal.pone.0082986 (PMC3888378; doi:10.1371/journal.pone.0082986)
Supplement: Table S1 — Recommendations for the Management of Osteoarthritis. ACR: American College of Rheumatology; EULAR: The European League against rheumatism; NICE: National Institute for health and Clinical Excellence; OARSI: Osteoarthritis Research Society International; PGrip: People Getting a Grip on Arthritis RACGP: Royal Australian College of General Practitioners TENS: Transcutaneous Electric Nerve Stimulation. (DOCX) [file pone.0082986.s001.docx]

| **Intervention** | AAOS | ACR | Eular | Eular | Kjeken | NICE | OARSI | Ottawa | Peter | Philadephia | RACGP | Roddy |
| --- | --- | --- | --- | --- | --- | --- | --- | --- | --- | --- | --- | --- |
|  |  |  |  |  | et al. |  |  | Panel | et al. | Panel |  | et al. |
|  | [26] | [27] | [28-30] | [31] | [32] | [33] | [34] | [35-39] | [42] | [40-41] | [44] | **[**43] |
| Electrotherapy | | | | | | | | | | | | |
| Low intensity laser therapy |  |  | Recommended |  |  |  | Strongly Recommended |  | Weak Evidence |  | Weak Evidence |  |
| Pulsed electromagnetic field |  |  | Recommended |  |  |  |  |  | Insufficient Evidence | Weak Evidence |  |  |
| Pulsed electrical stimulation of muscle |  |  | Recommended |  |  |  |  |  |  |  |  |  |
| TENS (high frequencies) |  | Recommended | Recommended |  |  |  | Strongly Recommended | Strongly Recommended |  | Strongly Recommended | Weak Evidence |  |
| TENS (low frequencies) |  | Recommended | Recommended |  |  |  | Strongly Recommended | Strongly Recommended |  | Strongly Recommended | Weak Evidence |  |
| Therapeutic Ultrasound |  |  | Recommended |  |  |  |  | Strongly Recommended | Weak Evidence | Weak Evidence | Weak Evidence |  |
|  |  |  | for hip & knee |  |  |  |  | Combined with exercise |  |  |  |  |
|  |  |  | Weakly Recommended |  |  |  |  |  |  |  |  |  |
|  |  |  | for hand |  |  |  |  |  |  |  |  |  |
| **Other Interventions** | | | | | | | | | | | | |
| Acupuncture | Weak Evidence | Recommended | Recommended |  |  |  | Strongly Recommended | Recommended |  |  | Weak Evidence |  |
| Assistive devices |  | Recommended | Recommended | Recommended |  | Recommended | Weak Evidence |  |  |  |  |  |
| Balneotherapy/ Spa Sauna |  |  | Recommended |  |  |  | Strongly Recommended | Strongly recommended | Weak Evidence |  |  |  |
| CAT (includes massage) |  |  |  |  |  |  | Strongly Recommended |  | Weak Evidence |  | Weak Evidence |  |
| Foot Orthoses and insoles |  | Recommended | Recommended (knee) |  |  |  | Strongly Recommended | Strongly Recommended |  |  | Recommended |  |
| Heat/Cryotherapy |  | Recommended | Recommended (hand) |  |  | Recommended | Strongly Recommended | Recommended | Weak Evidence |  | Weak Evidence |  |
|  |  |  |  |  |  |  |  | for ice massage |  |  |  |  |
| Hydrotherapy |  |  |  |  |  |  | Strongly Recommended |  |  |  |  |  |
| Joint Protection |  | Recommended |  |  |  |  | Strongly Recommended |  |  |  |  |  |
| Knee brace |  |  | Recommended |  |  |  | Strongly Recommended |  |  |  | Recommended |  |
| Leech Therapy |  |  |  |  |  |  |  |  |  |  | Insufficient Evidence |  |
| Magnetic Bracelets |  |  |  |  |  |  |  |  |  |  | Weak Evidence |  |
| Manual therapy combined to exercises |  | Recommended |  |  |  |  | Strongly Recommended |  | Recommended |  |  |  |
| Paraffin wax application |  |  | Strongly Recommended |  |  |  |  |  |  |  |  |  |
| Patellar Taping | Recommended | Recommended | Recommended |  |  |  |  |  | Recommended |  | Weak Evidence |  |
| Splinting-orthoses |  | Recommended | Recommended |  |  |  |  | Recommended (Thumb) |  |  |  |  |
| Patient Education | | | | | | | | | | | | |
| Patient Education | Recommended | Recommended | Strongly Recommended | Recommended |  | Recommended | Strongly Recommended | Strongly Recommended | Recommended |  | Weak Evidence | Strongly Recommended |
| Patient Education + Exercise |  |  |  |  |  |  | Strongly Recommended | Strongly Recommended |  |  |  | Strongly Recommended |
| Team Approach | | | | | | | | | | | | |
| Multidisciplinary Team Approach |  |  |  |  |  |  |  |  |  |  | Weak Evidence |  |
| Physiotherapy |  |  |  |  |  |  |  |  |  |  | Weak Evidence |  |
| **Therapeutic Exercises** | | | | | | | | | | | | |
| Aquatics |  | Strongly Recommended | Strongly Recommended |  |  |  | Recommended |  |  |  | Weak Evidence |  |
| Aerobic Exercises (AE) | Strongly Recommended | Strongly Recommended | Strongly Recommended | Recommended |  |  | Strongly Recommended | Strongly Recommended | Recommended |  |  | Strongly Recommended |
| Behavioural Approach + Exercise |  | Recommended |  | Recommended |  |  | Strongly Recommended | Strongly Recommended |  |  | Weak Evidence | Strongly Recommended |
| Functional Activity |  |  |  |  | Weak Evidence |  |  | Strongly Recommended |  | Strongly Recommended | Recommended |  |
| Dynamic Exercises | Weak Evidence |  |  |  | Strongly Recommended |  |  | Strongly Recommended |  |  |  |  |
| Group Exercise |  |  |  |  |  |  |  | Strongly Recommended |  |  |  | Strongly Recommended |
| Home Exercise |  |  | Strongly Recommended |  |  |  |  | Strongly Recommended |  |  |  | Strongly Recommended |
| Individualized Exercise |  |  |  |  |  |  |  | Strongly Recommended |  |  |  | Strongly Recommended |
| Patient Centered Approach |  |  |  |  |  |  |  |  |  |  |  | Weak Evidence |
| Range of Motion (ROM) |  |  | Strongly Recommended |  | Weak Evidence |  |  | Strongly Recommended |  |  |  |  |
| Stretching |  |  |  |  | Weak Evidence |  |  |  |  | Strongly Recommended |  |  |
| Strengthening Exercise (SE) |  | Strongly Recommended |  | Recommended | Strongly Recommended |  | Strongly Recommended | Strongly Recommended |  | Strongly Recommended |  | Strongly Recommended |
| Whole body exercises |  | Recommended |  |  |  |  |  | Strongly Recommended |  |  | Weak Evidence |  |
| (eg. Tai Chi and Yoga) |  |  |  |  |  |  |  |  |  |  |  |  |
| **Weight Management** | | | | | | | | | | | | |
| Control of weight | Strongly Recommended | Strongly Recommended | Recommended | Recommended |  | Recommended | Strongly Recommended | Strongly Recommended |  |  | Recommended |  |
| Diet |  |  |  |  |  |  |  | Strongly Recommended |  |  |  |  |
| Diet and Physical |  |  |  |  |  |  |  | Strongly Recommended |  |  |  |  |
| Physical Activity |  |  |  |  |  |  |  | Strongly Recommended |  |  |  |  |

**Table S1. Recommendations for the Management of Osteoarthritis**

CAT:Complementary and alternative therapies; ACR: American College of Rheumatology; EULAR: The European League against rheumatism; NICE: National Institute for health and Clinical Excellence; OARSI: Osteoarthritis Research Society International; PGrip: People Getting a Grip on Arthritis RACGP: Royal Australian College of General Practitioners TENS: Transcutaneous Electric Nerve Stimulation
