# Supplementary material for: A Systematic Critical Appraisal for Non-Pharmacological Management of Osteoarthritis Using the Appraisal of Guidelines Research and Evaluation II Instrument
Source: PLoS One. 2014 Jan 10;9(1):e82986. doi: 10.1371/journal.pone.0082986 (PMC3888378; doi:10.1371/journal.pone.0082986)
Supplement: Figure S1 — Prisma flow diagram of included CPGs. (DOC) [file pone.0082986.s002.doc]

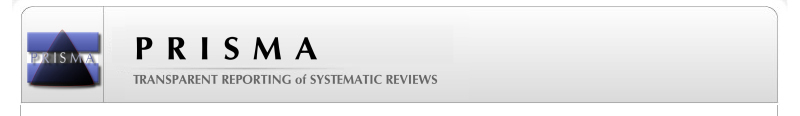
**Figure S1. PRISMA 2009 Flow Diagram**

**Screening**

**Included**

**Eligibility**

**Identification**

Records identified through database searching
(n=1136)

Additional records identified through other sources (hand search) (n=2)

(n=
(n =2)

Records after duplicates removed
(n=827)

Records screened
(n=40)

Records excluded based on abstract and title
(n =787)

Full-text articles assessed for eligibility
(n =18)

Full-text articles excluded, with reasons
(n =22):

- Not able to retrieve the guideline (n=6)
- CPGs did not contain a grading system (n=3)
- Considered only pharmacological interventions (n=3)
- Not a clinical practice guideline (n=9)
- Based on another clinical practice guideline already included (n=1).

CPGs included
(n = 17)
