# Supplementary material for: A Systematic Critical Appraisal for Non-Pharmacological Management of Osteoarthritis Using the Appraisal of Guidelines Research and Evaluation II Instrument
Source: PLoS One. 2014 Jan 10;9(1):e82986. doi: 10.1371/journal.pone.0082986 (PMC3888378; doi:10.1371/journal.pone.0082986)
Supplement: Appendix S2 — Literature Search Strategy. (DOCX) [file pone.0082986.s004.docx]

Appendix S2: Literature Search Strategy

Databases :

Embase  (n=715), MEDLINE (n= 226), CINAHL (n=170) & AMED (n=25).

**Limits: English literature, 2001-2013**

1. exp arthritis, rheumatoid/ (94715)
2. felty* syndrome.tw. (655)
3. caplan* syndrome.tw. (105)
4. (sicca adj2 syndrome).tw. (712)
5. still* disease.tw. (1492)
6. bechterew* disease.tw. (364)
7. rheumatoid nodule.tw. (226)
8. (arthritis adj2 rheumat*).tw. (72567)
9. exp osteoarthritis/ (39346)
10. osteoarthritis.tw. (30982)
11. osteoarthrosis.tw. (2760)
12. degenerative arthritis.tw. (964)
13. rheumatoid arthritis.tw. (71844)
14. rheumatism.tw. (8207)
15. rheumatoid.tw. (82277)
16. ankylosing spondylitis.tw. (8524)
17. arthrosis.tw. (4121)
18. sjogren*.tw. (10944)
19. or/1-18 (176791)
20. Splints/ or orthotic devices/ (11953)
21. (splint* or stent* or orthotics).tw. (66145)
22. 21 or 20 (73896)
23. (shoes* or cane* or crutches or walker* or assistive device* or self-help device* or toilet aid* or bath aid* or transfers or lifts or wheelchair* or wheel chair* or kitchen or beds or mattress* or ergonomic*).tw. (63354)
24. exp Self-Help Devices/ (7950)
25. brace/ or walking aid/ (4222)
26. Biomedical engineering/ or beds/ (13052)
27. or/23-26 (84040)
28. (cryotherapy or ice or hyperthermia or thermotherap* or hypothermia).tw. (64931)
29. (heat or hot or ice).tw. (185652)
30. cryotherapy.sh,tw. (6820)
31. (vapocoolant or phonophoresis).tw. (324)
32. exp hyperthermia, induced/ (21797)
33. (hypertherm* or thermotherapy).tw. (26892)
34. (fluidotherapy or compression).tw. (67429)
35. cryoanesthesia/ (30)
36. heat/tu (2649)
37. or/28-36 (313589)
38. (Conservation adj energy).tw. (6)
39. (fatigue or rest).tw. (148657)
40. fatigue/ (17324)
41. or/38-40 (154810)
42. exp electric stimulation therapy/ (53560)
43. ((electric$ adj1 nerve) or (electric$ adj1 therapy)).tw. (2325)
44. (electric$ adj (stimulation or muscle)).tw. (37957)
45. (electrostimulation or electroanalgesia or tens or altens or electroacupuncture or iontophoresis).tw. (16086)
46. or/42-45 (99005)
47. Patient Education/ (65821)
48. patient educat$.tw. (10444)
49. 48 or 47 (70501)
50. exp Acupuncture Therapy/ (14940)
51. (acupuncture or electro-acupuncture or electroacupuncture).tw. (14319)
52. Acupressure.tw. (500)
53. or/50-52 (18243)
54. (intensit$ adj3 rehabilitation).tw. (142)
55. exp Physical Therapy Modalities/ (113700)
56. (intensit$ or strength).tw. (384861)
57. 56 and 55 (10176)
58. exp exercise therapy/ (26137)
59. exercise$.tw. (176648)
60. 59 or 58 (188657)
61. exp Musculoskeletal Manipulations/ (11305)
62. (manual therap$ or manipulation$).tw. (69934)
63. 61 or 62 (78743)
64. 22 or 27 or 37 or 41 or 46 or 49 or 53 or 57 or 60 or 63 (1006571)
65. 19 and 64 (10726)
66. (systematic: review: or systematic: overview).tw. (39933)
67. guideline*.ti,ab. or practice guideline.pt. (169020)
68. 65 and (66 or 67) (419)
