# Supplementary material for: A Systematic Critical Appraisal for Non-Pharmacological Management of Osteoarthritis Using the Appraisal of Guidelines Research and Evaluation II Instrument
Source: PLoS One. 2014 Jan 10;9(1):e82986. doi: 10.1371/journal.pone.0082986 (PMC3888378; doi:10.1371/journal.pone.0082986)
Supplement: Appendix S3 — The Eligibility Criteria. (DOCX) [file pone.0082986.s005.docx]

| **The Inclusion Criteria** | **Rationale for the Inclusion and Exclusion Criteria** | **The Exclusion Criteria.** |
| --- | --- | --- |
| 1. Published CPGs must be peer reviewed. | 1. Web based CPGs are harder to access after certain duration and can be unreliable. | 1. CPGs that are solely web based. |
| 1. The CPGs must identify the authors. | 1. The Authors name must be clearly indicated for each CPG. | 1. CPGs that are authored as a professional group/association. |
| 1. CPGs must include a reference list. | 1. CPGs must be based on scientific data and thus provide references. | 1. CPGs that exclude the reference list. |
| 1. The CPG must use a grading system to evaluate the level of evidence for each recommendation. | 1. The recommendations for the CPGs should be issued on the basis of the body of evidence relevant to the research question. | 1. CPGs that provide recommendations without a grading system. |
| 1. CPGs based on systematic reviews. | 1. Systematic reviews offered a less biased recommendation (avoiding potential publication biases). | 1. CPGs not clearly based on systematic reviews. |
| 1. The CPGs must be written in English. | 1. The CPGs must be written in English for the comprehension of the guidelines and AGREE II content. | 1. CPGs written in languages other than English were excluded. |
| 1. The CPGs must be current (2001-2012) and must mention a release date. | 1. Only recent CPG’s were included. The presence of release date was necessary to ensure currency. | 1. CPGs that were published before 2000. |
| 1. CPGs must involve participants’ ≥18 years of age. | 1. Paediatrics needs more specific recommendations. | 1. CPGs with participants <18 years of age. |
| 1. CPGs must involve comparative controlled studies which examined effectiveness of specific non-pharmacological interventions in rheumatology. | 1. Professional associations and organizations often publish “guidelines” pertaining to ethics or practice standards and not to physical rehabilitation interventions themselves. Only guidelines referring to physical interventions were selected. | 1. CPGs that looked only at pharmaceutical or surgical interventions. |
| 1. CPGs must be relevant to Osteoarthritis (OA) in the upper and lower extremities of the body. | 1. OA is one of the most prevalent diseases in Rheumatology. OA in the spine can involve for instance neurological condition, which requires different interventions. | 1. CPGs that looked at other rheumatologic conditions. |

**Appendix S3. The Eligibility Criteria**
