# Supplementary material for: A Systematic Critical Appraisal for Non-Pharmacological Management of Osteoarthritis Using the Appraisal of Guidelines Research and Evaluation II Instrument
Source: PLoS One. 2014 Jan 10;9(1):e82986. doi: 10.1371/journal.pone.0082986 (PMC3888378; doi:10.1371/journal.pone.0082986)
Supplement: Appendix S4 — Raw Data. (DOCX) [file pone.0082986.s006.docx]

| AGREE II items | AAOS  [26] | | ACR  [27] | | Eular  [28-30] | | Eular  [31] | | Kjenken  [32] | | NICE  [33] | | OARSI  [34] | | Ottawa Panel  [35-39] | | Peter  et al.  [42] | | Philadelphia  Panel  [40-41] | | RACGP  [44] | | Roddy  et al.  [43] | |
| --- | --- | --- | --- | --- | --- | --- | --- | --- | --- | --- | --- | --- | --- | --- | --- | --- | --- | --- | --- | --- | --- | --- | --- | --- |
|  | Domain 1. Scope and Purpose | | | | | | | | | | | | | | | | | | | | | | | |
| 1 | 7 | 7 | 6 | 7 | 5 | 4 | 6 | 6 | 7 | 7 | 5 | 5 | 5 | 7 | 7 | 7 | 6 | 6 | 7 | 7 | 7 | 7 | 6 | 7 |
| 2 | 4 | 6 | 6 | 6 | 2 | 2 | 4 | 6 | 4 | 5 | 6 | 4 | 3 | 5 | 7 | 7 | 4 | 6 | 7 | 7 | 3 | 6 | 4 | 6 |
| 3 | 4 | 6 | 6 | 7 | 2 | 2 | 2 | 2 | 2 | 1 | 6 | 4 | 2 | 4 | 6 | 7 | 2 | 2 | 6 | 7 | 5 | 7 | 5 | 5 |
|  | Domain 2. Stakeholder Involvement | | | | | | | | | | | | | | | | | | | | | | | |
| 4 | 4 | 7 | 7 | 7 | 4 | 7 | 6 | 7 | 3 | 1 | 6 | 7 | 5 | 7 | 6 | 7 | 5 | 7 | 6 | 7 | 5 | 7 | 5 | 7 |
| 5 | 1 | 3 | 4 | 2 | 1 | 1 | 5 | 5 | 1 | 1 | 7 | 5 | 3 | 1 | 4 | 2 | 3 | 3 | 1 | 2 | 2 | 5 | 1 | 1 |
| 6 | 7 | 6 | 6 | 6 | 1 | 2 | 6 | 5 | 5 | 2 | 7 | 6 | 7 | 7 | 6 | 7 | 5 | 7 | 7 | 7 | 7 | 7 | 4 | 2 |
|  | Domain 3. Rigour of Development | | | | | | | | | | | | | | | | | | | | | | | |
| 7 | 4 | 7 | 5 | 6 | 7 | 7 | 7 | 7 | 4 | 5 | 3 | 1 | 6 | 7 | 6 | 6 | 5 | 5 | 5 | 6 | 7 | 7 | 5 | 5 |
| 8 | 6 | 6 | 5 | 6 | 6 | 5 | 5 | 5 | 5 | 5 | 7 | 5 | 7 | 7 | 7 | 7 | 3 | 2 | 7 | 7 | 6 | 6 | 5 | 4 |
| 9 | 4 | 6 | 5 | 6 | 2 | 4 | 4 | 5 | 3 | 2 | 2 | 7 | 2 | 4 | 7 | 5 | 3 | 2 | 5 | 5 | 5 | 3 | 4 | 5 |
| 10 | 6 | 7 | 7 | 7 | 4 | 7 | 5 | 2 | 6 | 7 | 2 | 7 | 6 | 6 | 4 | 7 | 4 | 4 | 7 | 7 | 3 | 7 | 6 | 7 |
| 11 | 4 | 7 | 5 | 7 | 7 | 5 | 6 | 6 | 4 | 1 | 7 | 7 | 7 | 7 | 5 | 5 | 5 | 5 | 2 | 2 | 6 | 7 | 4 | 7 |
| 12 | 6 | 7 | 5 | 7 | 5 | 3 | 7 | 7 | 6 | 7 | 7 | 7 | 7 | 7 | 5 | 5 | 6 | 7 | 7 | 7 | 6 | 7 | 6 | 7 |
| 13 | 6 | 7 | 2 | 3 | 1 | 2 | 2 | 1 | 4 | 5 | 5 | 3 | 4 | 5 | 3 | 5 | 3 | 3 | 7 | 7 | 6 | 7 | 1 | 1 |
| 14 | 4 | 6 | 2 | 2 | 1 | 2 | 1 | 1 | 1 | 1 | 7 | 6 | 4 | 5 | 1 | 1 | 1 | 1 | 1 | 1 | 5 | 6 | 1 | 1 |
|  | Domain 4. Clarity of Presentation | | | | | | | | | | | | | | | | | | | | | | | |
| 15 | 5 | 7 | 5 | 6 | 5 | 5 | 6 | 6 | 6 | 6 | 5 | 6 | 5 | 7 | 5 | 7 | 6 | 7 | 5 | 5 | 5 | 7 | 6 | 6 |
| 16 | 7 | 5 | 7 | 7 | 7 | 7 | 6 | 6 | 5 | 7 | 5 | 6 | 7 | 7 | 5 | 5 | 7 | 7 | 7 | 7 | 7 | 7 | 6 | 6 |
| 17 | 7 | 7 | 7 | 7 | 7 | 7 | 7 | 7 | 7 | 6 | 6 | 7 | 7 | 7 | 4 | 4 | 7 | 7 | 5 | 5 | 7 | 7 | 7 | 7 |
|  | Domain 5. Applicability | | | | | | | | | | | | | | | | | | | | | | | |
| 18 | 1 | 1 | 1 | 2 | 1 | 1 | 1 | 1 | 1 | 1 | 2 | 4 | 3 | 2 | 4 | 2 | 1 | 1 | 6 | 6 | 4 | 1 | 1 | 1 |
| 19 | 1 | 1 | 1 | 2 | 1 | 1 | 1 | 1 | 1 | 1 | 5 | 5 | 4 | 2 | 1 | 1 | 1 | 1 | 2 | 2 | 5 | 4 | 1 | 1 |
| 20 | 1 | 1 | 1 | 2 | 5 | 5 | 1 | 1 | 1 | 1 | 7 | 4 | 4 | 2 | 2 | 1 | 1 | 1 | 1 | 1 | 1 | 1 | 1 | 1 |
| 21 | 1 | 1 | 2 | 2 | 1 | 1 | 1 | 1 | 1 | 1 | 5 | 3 | 2 | 2 | 1 | 1 | 1 | 1 | 1 | 1 | 1 | 1 | 1 | 1 |
|  | Domain 6. Editorial Independence | | | | | | | | | | | | | | | | | | | | | | | |
| 22 | 3 | 6 | 4 | 3 | 3 | 1 | 3 | 3 | 3 | 3 | 3 | 3 | 3 | 4 | 5 | 5 | 3 | 4 | 3 | 3 | 3 | 6 | 3 | 3 |
| 23 | 6 | 6 | 2 | 2 | 1 | 1 | 3 | 3 | 3 | 3 | 2 | 2 | 7 | 7 | 2 | 1 | 1 | 1 | 1 | 1 | 2 | 1 | 3 | 3 |
|  | Overall Assessment | | | | | | | | | | | | | | | | | | | | | | | |
| A^1^ | 5 | 6 | 5 | 5 | 4 | 4 | 4 | 5 | 4 | 4 | 5 | 6 | 4 | 5 | 5 | 6 | 4 | 4 | 5 | 5 | 5 | 7 | 6 | 6 |
| B^2^ | Y | Y | Y | Y | M | M | Y | Y | M | M | Y | Y | M | Y | Y | Y | M | M | Y | Y | Y | Y | Y | Y |

ACR: American College of Rheumatology; EULAR: The European League against rheumatism; NICE: National Institute for health and Clinical Excellence; OARSI: Osteoarthritis Research Society International; RACGP: Royal Australian College of General Practitioners

**LEGEND**

 Evaluator 1 (KTA & LB)

Evaluator 2 (PR)

^1^A: Rate the overall quality of this guideline.

^2^B: I would recommend this guideline for use.

Y: Yes

M: Yes, with modification

N: No
